# Toward Low-Cost and Sustainable Supercapacitor Electrode Processing: Simultaneous Carbon Grafting and Coating of Mixed-Valence Metal Oxides by Fast Annealing

**DOI:** 10.3389/fchem.2019.00025

**Published:** 2019-02-06

**Authors:** Keyvan Malaie, Mohammad Reza Ganjali, Francesca Soavi

**Affiliations:** ^1^Center of Excellence in Electrochemistry, School of Chemistry, College of Science, University of Tehran, Tehran, Iran; ^2^Biosensor Research Center, Endocrinology and Metabolism Molecular-Cellular Sciences Institute, Tehran University of Medical Sciences, Tehran, Iran; ^3^Department of Chemistry “Giacomo Ciamician”, Alma Mater Studiorum-Università di Bologna, Bologna, Italy

**Keywords:** iron oxide, manganese oxide, carbon black, nanocomposite, thermal annealing, pseudocapacitor

## Abstract

There is a rapid market growth for supercapacitors and batteries based on new materials and production strategies that minimize their cost, end-of-life environmental impact, and waste management. Herein, mixed-valence iron oxide (FeO_x_) and manganese oxide (Mn_3_O_4_) and FeO_x_-carbon black (FeO_x_-CB) electrodes with excellent pseudocapacitive behavior in 1 M Na_2_SO_4_ are produced by a one-step thermal annealing. Due to the *in situ* grafted carbon black, the FeO_x_-CB shows a high pseudocapacitance of 408 mF cm^−2^ (or 128 F g^−1^), and Mn_3_O_4_ after activation shows high pseudocapacitance of 480 mF cm^−2^ (192 F g^−1^). The asymmetric supercapacitor based on FeO_x_-CB and activated-Mn_3_O_4_ shows a capacitance of 260 mF cm^−2^ at 100 mHz and a cycling stability of 97.4% over 800 cycles. Furthermore, due to its facile redox reactions, the supercapacitor can be voltammetrically cycled up to a high rate of 2,000 mV s^−1^ without a significant distortion of the voltammograms. Overall, our data indicate the feasibility of developing high-performance supercapacitors based on mixed-valence iron and manganese oxide electrodes in a single step.

## Introduction

Supercapacitors are attracting increasing attention in today's fast-growing electronics industry. They can provide high power density and stability because they utilize fast charge/discharge processes at the electrode/electrolyte interface. These processes are of two types: ion adsorption/desorption at the interface (i.e., EDLC) and fast faradaic electrode reactions that are exploited in pseudocapacitors (Brousse et al., 2015).

There is a growing interest in pseudocapacitors due to their potential for accessing higher energy densities than those of the traditional EDLC supercapacitors. Their fast redox reactions bring about charge storage capability in the bulk of materials higher than the EDLC that stores charge by a surface electrostatic process (Lukatskaya et al., 2017). Iron oxide and manganese oxide are examples of materials with pseudocapacitive behavior (Simon et al., 2017). They are universally abundant, environmentally benign, inexpensive, electrochemically-active in non-corrosive neutral electrolytes, and completely safe after disposal of the supercapacitor (Dyatkin et al., 2013).

In addition, iron and manganese oxides have complementary working potential windows, making them appealing for developing high-voltage aqueous asymmetric supercapacitors. Probably their only undesirable property is their low electrical conductivity that results in high IR drops at high charge/discharge rates. However, the mixed-valance iron or manganese oxides (i.e., spinel oxides) have a better conductivity. For example, Fe_3_O_4_ has a very high electric conductivity of 2 × 10^4^ S m^−1^ at 25°C (Malaie et al., 2018).

Recent research is focused on development of supercapacitor materials and processes that enable low cost and low end-of-life environmental impact and easy waste management on a large production scale (Dyatkin et al., 2013). Therefore, non-precious metal oxides like FeO_x_ and MnOx-based electrodes are attracting much attention. Pseudocapacitors based on Fe_3_O_4_ as the negative electrode and MnO_2_ as the positive electrode have been synthesized by various methods (Brousse and Bélanger, 2003; Yang et al., 2014; Gund et al., 2015). These methods include chemical vapor deposition (CVD), electrodeposition, hydrothermal, and sol-gel. However, there are several concerns that make these synthesis strategies unappealing for scaling-up applications. First, the films grown by the CVD, electrodeposition and hydrothermal methods provide a loading mass in the range of tens of μg cm^−2^ to a few mg cm^−2^, while commercial supercapacitors require 8–10 mg cm^−2^ to give a practical areal capacitance (Balducci et al., 2017; Song et al., 2017). Second, for the preparation of hybrid materials, these methods usually utilize prolonged and multi-step processes, elevated temperatures, complex instruments, and special precursor materials (Qian et al., 2012). Third, the conductive additives (e.g., graphene, CNT, …) that are usually composited with redox materials to reduce electrical resistance and enhance utilization of redox sites are very expensive for large-scale production; but carbon black materials that are easily obtained by carbonization of organic materials are significantly cheaper. On the other hand, rapid preparation of nanomaterials especially at elevated temperatures usually results in the enlargement and aggregation of the particles because under these conditions the growth of the particles is hardly controllable. Therefore, there is a need for general electrode processing methods that afford high-areal capacitance electrodes with time and cost efficiency.

Herein, for the first time, we report a novel synthesis approach to develop green supercapacitors based on binder-free, non-precious metal oxides electrodes, that is, a fast thermal annealing (FTA) method for the preparation of pseudocapacitor electrodes based on amorphous iron oxide-carbon black (FeO_x_-CB) and Mn_3_O_4x_ with high areal capacitances. Our method unifies the following three common steps of electrode preparation into a single step: (i) synthesis of the metal oxides, (ii) composite material processing with a carbon conductive additive, and (iii) coating on the current collector. We show that CB (or other conductive elements) can be *in situ* composited with the metal oxides and simultaneously coated on the nickel foam without employing any binder, which improves the electrochemical performance of the pseudocapacitor substantially by reducing the electrical resistance and promoting charge transfer rate. FTA is carried out at moderately low temperatures that reduces the energy cost of electrode production at large scale. It also requires a minimum amount of materials (i.e., only a metal nitrate in 5–10 ml of ethylene glycol as solvent) to prepare the electrodes; therefore, the waste produced during the electrode processing is also very small. Finally, our method paves the way toward new electrode manufacturing processes that exclude the use of binders, like Teflon and PVDF. It avoids the use of binders and solvents required to cast active materials on current collectors, that represents an additional value for green and low-energy demanding processes.

## Experimental

Ferric nitrate nonahydrate (Fe(NO_3_)_3_.9H_2_O, 99%), manganese nitrate hexahydrate (Mn(NO_3_)_2_.6H_2_O, 99%), ethylene glycol (98%), and sodium sulfate nonahydrate (Na_2_SO_4_.9H_2_O, 99.99%) were purchased from Sigma-Aldrich Company. Carbon black (Super-P®, BET 65.5 m^2^ g^−1^) was purchased from Erachem Comilog Company. Nickel foam was purchased from Changsha Lyrun Material Company (Shangsha, China).

### Preparation of FeO_x_-CB/Ni Foam

Nickel foam was cut into circular pieces with a diameter of 0.9 cm (area: 0.64 cm^2^). Then they were cleaned with 10% HCl and deionized water, sequentially. The FeO_x_-CB was synthesized directly onto the Ni foam. In a typical synthesis, 0.5 mmol Fe(NO_3_)_3_.9H_2_O and 10 mg CB were dissolved in 10 mL of ethylene glycol by vigorous stirring. The Ni foam was immersed in the solution and the solution was heated on a hot plate to 150°C for 20 min during which the solution is quickly dehydrated. Then it was immediately heated to 300°C for 5 min, resulting in the deposition of FeO_x_-CB on the Ni foam after a brief exhaust of voluminous smoke. Finally, the FeO_x_-CB/Ni foam was taken out and washed successively with water and ethanol. The FeO_x_ was also prepared by the same method but without the CB.

### Preparation of Mn_3_O_4_/Ni Foam

The Mn_3_O_4_/Ni foam was prepared according to the method in section Preparation of FeO_x_-CB/Ni Foam. Then, it was electrochemically activated by 200 successive voltammetric cycles in 1 M Na_2_SO_4_, and named a-Mn_3_O_4_.

### Physical Characterization

The materials were characterized by X-ray diffraction (XRD) on a Philips PW-1730 X-ray diffractometer using Cu Kα radiation λ = 1.5405 Å. Thermal gravimetric analysis (TGA) measurements were carried out in oxygen atmosphere. The samples were analyzed on a platinum pan under an oxygen flow rate of 60.0 mL/min with a temperature ramp of 10°C/min up to 600°C. Surface morphology and elemental composition of the materials were studied by the field emission scanning electron microscopy (FE-SEM) equipped with an energy dispersive X-ray spectrometer (EDS) on a Zeiss SIGMA VP. Transmission electron microscopy (TEM) images were obtained by using a Philips CM100.

### Electrochemical Measurements

The oxidation state and the stoichiometry of the iron oxide in FeO_x_-CB sample was determined based on a simple potentiometric redox titration of Fe(II) ions in the dissolved sample. Details can be found in section Preparation of FeO_x_-CB/Ni Foam, Supplementary Material. The electrodes were studied by cyclic voltammetry (CV), galvanostatic charge-discharge (GCD), and electrochemical impedance spectroscopy (EIS) using a potentiostat/galvanostat (PGSTAT M101, Metrohm Autolab B.V) in three-electrode and two-electrode configurations. For three-electrode measurements, Hg/Hg_2_Cl_2_ (3 M KCl), platinum coil, and FeO_x_-CB/Ni foam or a-Mn_3_O_4_/Ni foam were used as the reference electrode, counter electrode, and working electrode, respectively. EIS measurements were carried out in a frequency range from 100 kHz to 100 mHz with an AC potential of 10 mV. One molar of Na_2_SO_4_ was used as the electrolyte. The mass loading of the materials on the Ni foam was 3–4 mg cm^−2^.

Preliminary three-electrode studies were carried out using conventional glass electrochemical cells. For the full cell studies, T-shape Teflon Swagelok-type cells (BOLA) with a 100 μm separator and 0.64 cm^2^ electrode disks were used. The reference electrode was set in the middle of the cell to monitor each electrode potential during the supercapacitor cycling tests.

The electrode capacitance (C) was calculated from cyclic voltammetry by the slope of the linear part of the plots of the integrated current over time (upon CV discharge) vs. electrode potential. Electrode areal capacitance (F cm^−2^) and electrode specific capacitance (F g^−1^) were obtained by normalizing the capacitance to the electrode geometric area and to the mass of FeO_x_-CB or a-Mn_3_O_4_, respectively (Ni foam weight is excluded).

For measuring specific capacitance based on the GCD, the discharge curves were first fitted to a straight line, and then the capacitance was calculated from the reciprocal of the slope of electrode potential (for three-electrode set up) or cell voltage (for 2-electrode set up) vs. discharge capacity. More details on the calculation of the supercapacitor parameters (Capacitance, and energy/power density) and their formulas can be found in section Preparation of Mn_3_O_4_/Ni Foam, Supplementary Material.

## Results and Discussions

**Scheme 1:**
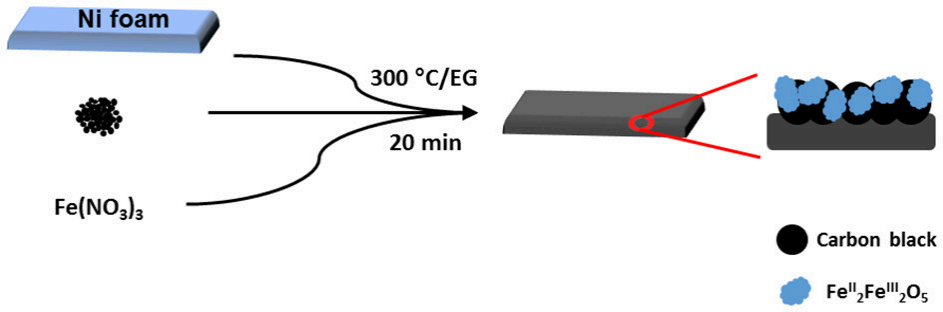
Fast thermal annealing of the electrode precursors on nickel foam in ethylene glycol.

A schematic representation of the electrode processing for iron oxide nanocomposite is shown in Scheme 1. The mechanism for FTA deposition of the metal oxides is proposed as follows. During the thermal annealing, M^n+^ ions are solvolysed and form metal alkoxides. Then, an exothermic flameless auto-combustion reaction between EG and ${\text{NO}}_{3}^{-}$ takes place, that raises the temperature further and drives the olation reaction of metal alkoxides to metal oxides. An overall reaction can be proposed as follows:

(1) $$\begin{array}{l} {\text{C}}_{2}{\text{H}}_{6}{\text{O}}_{2}+\text{3Fe}{\left({\text{NO}}_{3}\right)}_{3}\to {\text{Fe}}_{3}{\text{O}}_{4}+{\text{9NO}}_{2}+{\text{2CO}}_{2}+{\text{3H}}_{2}\text{O}+\text{Q} \end{array}$$

In the meantime, the ethylene glycol is also polymerized to polyglycolic acid (Takahashi et al., 2016) that can act as an internal binder in the carbon-metal oxides coated on the nickel foam.

### Structure and Morphology

**Figure 1:**
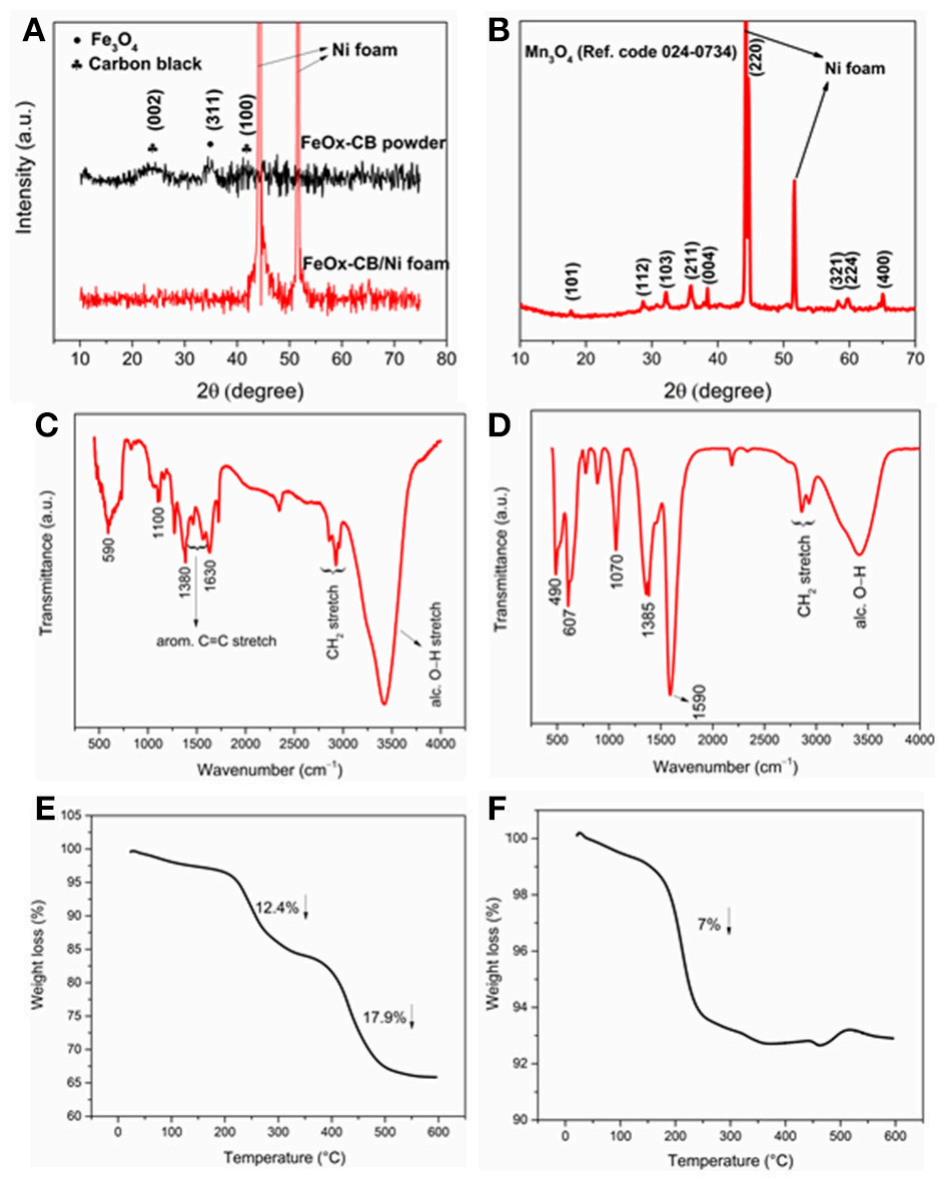
**(A)** XRD patterns of the FeO_x_-CB powder and coated on Ni foam, **(B)** XRD pattern of Mn_3_O_4_ coated on Ni foam, IR spectrum of FeO_x_-CB **(C)**, and Mn_3_O_4_ **(D)**. TGA plots of FeO_x_-CB **(E)** and Mn_3_O_4_ powders **(F)**.

Figure 1A shows the XRD patterns of the FeO_x_-CB powder and FeO_x_-CB/Ni foam. They show any a few slight peaks, signifying the rather amorphous nature of the nanocomposite. The XRD pattern of FeO_x_-CB powder shows a peak between 20 and 30° due to the (002) plane of graphitic carbon (Liu et al., 2010) and two other peaks at 35 and 42.5° due to the (311) and (400) planes of Fe_3_O_4_. Figure 1B shows the XRD pattern of the manganese oxide/Ni foam obtained by the FTA method. The reflections for the planes of (101), (112), (103), (211), (004), (220), (321), (324), and (400) are indexed to the tetragonal hausmannite structure of Mn_3_O_4_ [Ref. Code 24-0734] in agreement with other reported Mn_3_O_4_ compound (Dubal et al., 2010a). Figures 1C,D shows the IR spectra of the FeO_x_-CB and Mn_3_O_4_ samples. The peak at 590 cm^−1^ is due to Fe-O stretch in Fe_3_O_4_ (Figure 1C), and the two strong peaks at 490 and 607 cm^−1^ for Mn_3_O_4_ (Figure 1D) are due to coupling between Mn-O stretching vibrations at tetrahedral and octahedral sites (Tian et al., 2013). Both spectra share carbon-oxygen functional features including alcoholic hydroxyl stretch (3,420 cm^−1^), symmetric and asymmetric stretch of carboxylate (~1,590 and 1,385 cm^−1^), and C-O stretch (~1,070). Therefore, both materials are highly hydrophilic.

Figure 1E shows the TGA of the FeO_x_-CB sample. It shows two distinct weight losses at about 250 and 450°C. The first weight loss is due to the decomposition of carbon-oxygen functional groups such as –COH and –COOH that have survived the thermal annealing. Similar weight losses in TGA has been also reported for the decomposition of oxygen functional groups in graphene oxide (Wojtoniszak et al., 2012; Dehghanzad et al., 2016). The second weight loss at 450°C is due to the oxidation of the added carbon black in the nanocomposite (Lim et al., 2013; Zha et al., 2015; Li et al., 2017). Based on these two weight losses, the amount of total carbon content in the sample is 30.2 wt.%. Figure 1F shows the TGA of the Mn_3_O_4_ sample. As expected, it shows only one weight loss at around 225°C due to the decomposition of carbon-oxygen groups that account for 7.0 wt.% of the sample.

**Figure 2:**
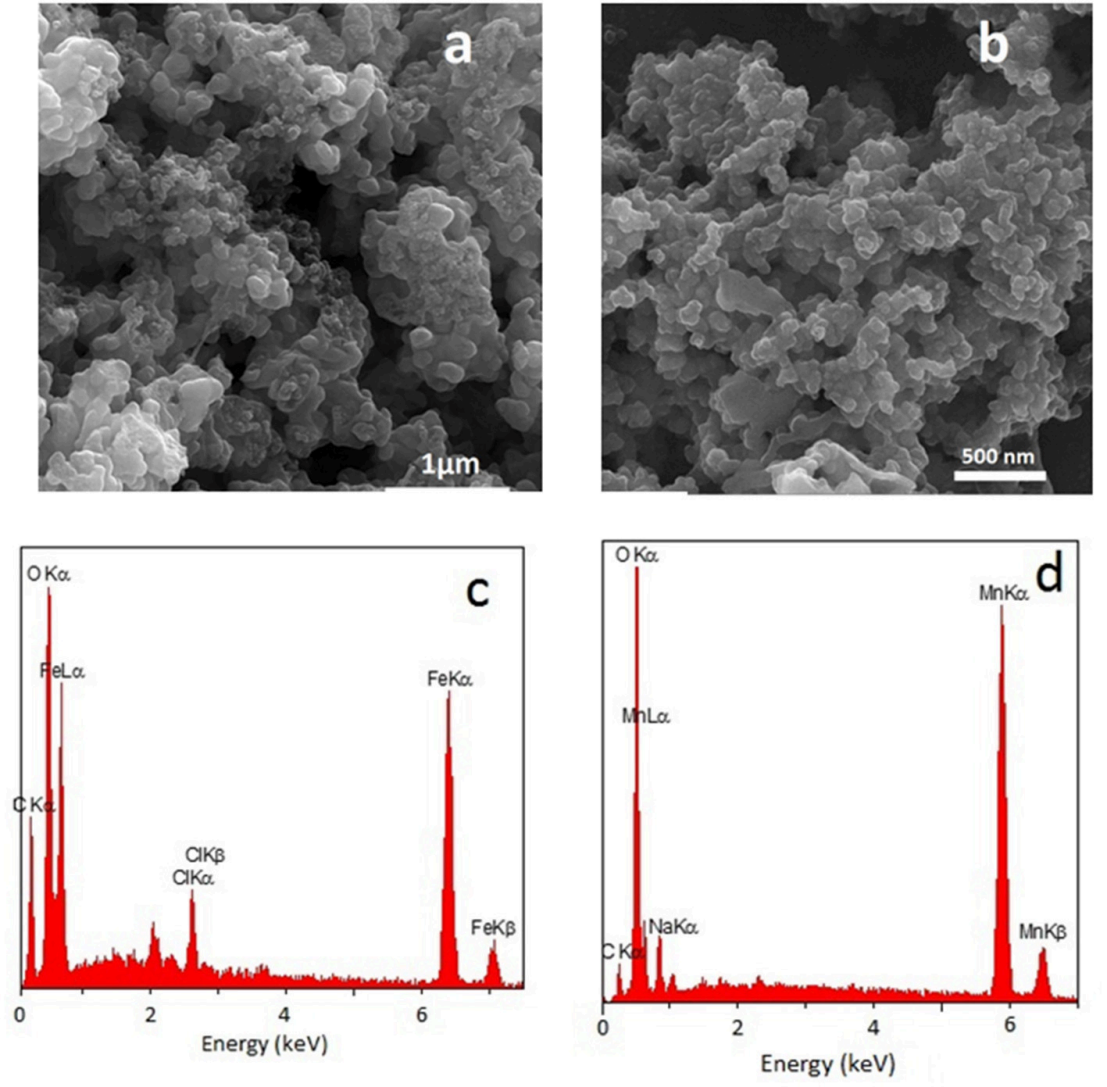
FESEM image and EDS spectrum of FeO_x_-CB containing 30 wt.% total carbon annealed at 300°C **(a,c)** and FESEM image and EDS spectrum of Mn_3_O_4_ **(b,d)**.

Figure 2a shows a representative FESEM image of the FeO_x_-CB sample. The FeO_x_-CB particles appear as interconnected nanospheres creating a macroporous surface structure. The FESEM image of the Mn_3_O_4_ (Figure 2b) shares similar morphological features. Figure 2c shows the EDS of the FeO_x_-CB. It confirms the presence of Fe, O, and C elements in the powder, and its elemental mapping analysis (Figure S1) shows that these elements are distributed quite homogeneously within the particles. According to the EDS, the carbon element accounts for 26.5 wt.% of the FeO_x_-CB sample (Table S1), agreeing with the total carbon content from the TGA (30.2 wt.%.). The molar ratio of O/Fe is almost three times higher than those of the known stoichiometric iron oxides (i.e., Fe_3_O_4_ or Fe_2_O_3_) (Table S1), confirming the presence of abundant hydrophilic oxygen-carbon groups in the sample. The EDS of Mn_3_O_4_ (Figure 2d) also confirms the presence of Mn, O, and partial amount of C from the burnt ethylene glycol.

**Figure 3:**
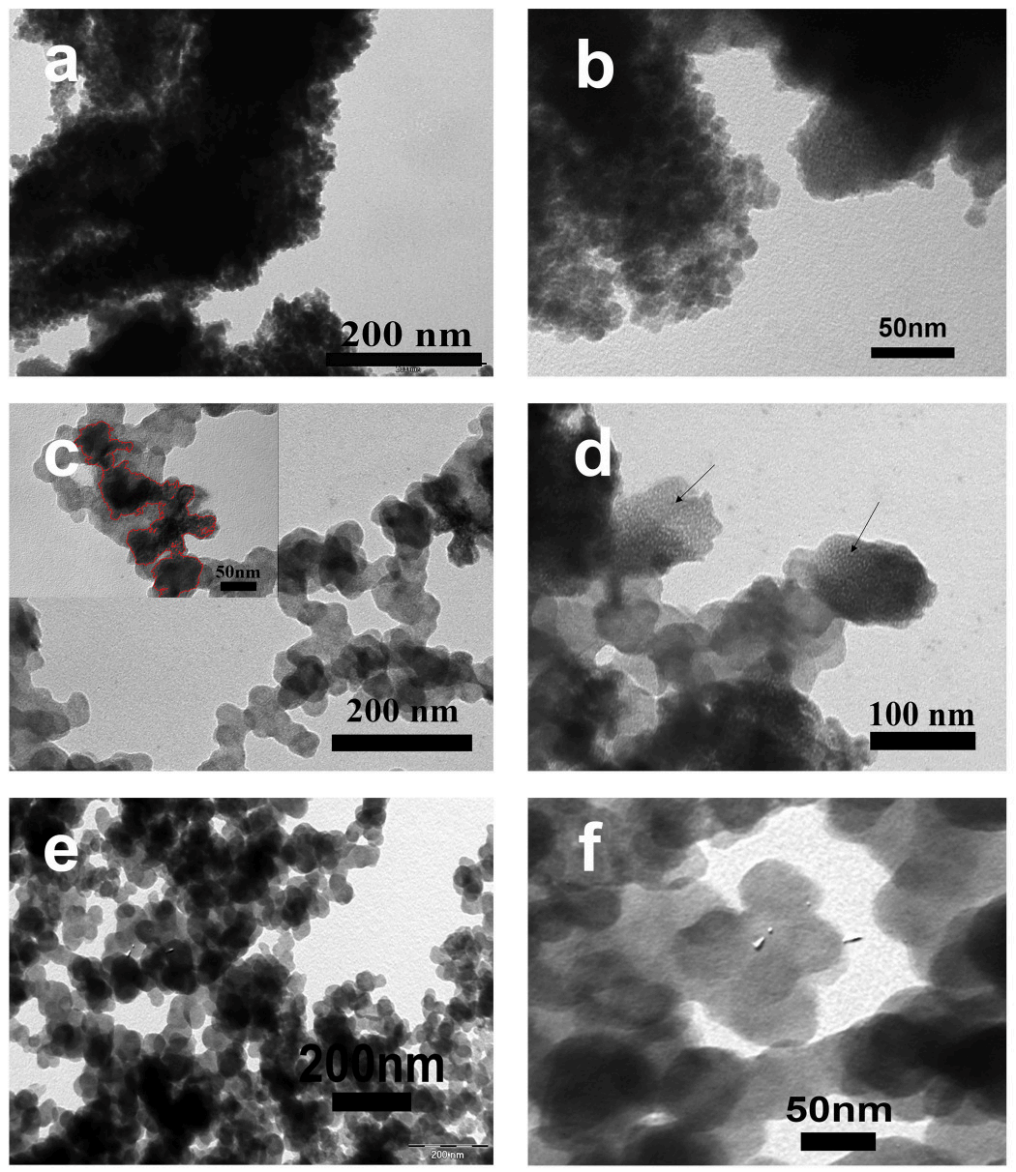
TEM images of FeO_x_ annealed at 300°C **(a,b)**, FeO_x_-CB containing 18 wt.% carbon black annealed at 300°C **(c,d)** (Inset in **c** shows FeO_x_ anchored on CB particles), and **(e,f)** pristine carbon black.

Figure 3 shows the TEM images of FeO_x_, FeO_x_-CB, and pristine CB. The TEM images of FeO_x_ (Figures 3a,b) show that FeO_x_ particles are highly aggregated without a specific shape. However, the TEM image of the FeO_x_-CB (Figure 3c) shows that FeO_x_ particles have been anchored on the CB particles leading to higher dispersity. The CB particles, in fact, have created a conducting network within the iron oxide particles. Furthermore, Figure 3d shows (meso) porous regions for FeO_x_-CB, as indicated by the arrows, that are favorable for facile ion diffusion. Figures 3e,f show the TEM images of pristine CB. It shows CB particles with an average diameter of 50 nm without any surface porosity.

### Electrochemical Studies

**Figure 4:**
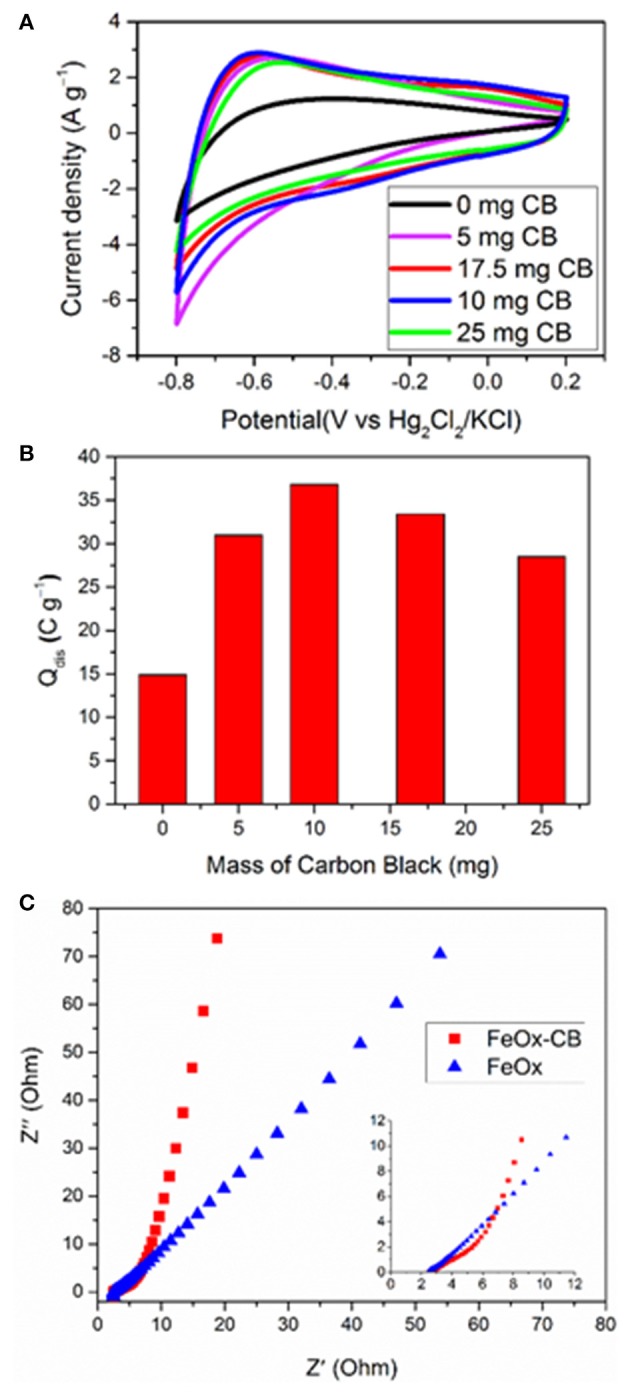
The effect of CB on the electrochemical performance of FeO_x_-CB with 18 wt.% carbon black in 1 M Na_2_SO_4_ **(A)** CV curves at 50 mV s^−1^ for FeO_x_-CB annealed at 300°C, **(B)** Discharge capacity (Q) measured based on the CV curves, **(C)** Nyquist plot (inset: maximized high-frequency region).

Before studying the electrochemical performance of the FeO_x_-CB/Ni foam electrode developed based on the FTA method, the oxidation state of the iron oxide and its stoichiometry were estimated by a simple method. A potentiometric redox titration of the dissolved iron oxide by permanganate revealed that the sample contains 24.3 wt.% Fe(II). A simple calculation revealed a stoichiometry of ${\text{Fe}}_{2}^{\text{II}}$${\text{Fe}}_{2}^{\text{III}}$O_5_ that is consistent with a mixture of FeO (wustite) and Fe_3_O_4_ (magnetite) (Figure S2 and section Electrochemical Studies in Supplementary Material). Figure 4A shows the CVs at 50 mV s^−1^ of the FeO_x−_CB electrodes synthesized from starting solutions containing different amounts of carbon black annealed at 300°C. The FeO_x_ without carbon black shows the lowest current densities. The samples containing carbon black show significant enhancements in their current densities along with more defined redox peaks. This effect can be explained as follows: the carbon black promotes the electrical contact among the FeO_x_ particles and the Ni foam and promotes charge transfer (Sayahi et al., 2014), therefore, it increases the utilization of the electroactive material. The redox peaks are around −0.4 and −0.8 V vs. Hg/Hg_2_Cl_2_ (cathodic peaks) and −0.6 and −0.1 V vs. Hg/Hg_2_Cl_2_ (anodic peaks). The underlying redox reactions are not fully known, but similar redox peaks have been also reported by Brousse et al. for iron oxide-carbon composites in Na_2_SO_4_ solution (Gao et al., 2014; Rebuttini et al., 2015). Figure 4B shows a plot of discharge Q, calculated based on the cyclic voltammograms vs. the pre-mixing weight of the carbon black. It shows that the discharge capacity Q normalized to the amount of FeO_x_-CB composite on the electrode reaches its highest value of 36 C g^−1^ when the pre-mixing weight of carbon black is 10 mg (about 18 wt.% of the FeO_x_-CB weight); therefore, this value was selected as the optimum amount of CB. Figure 4C compares the Nyquist plots of the FeO_x_ and the optimum FeO_x_-CB, demonstrating the beneficial effect of CB. Indeed, while for the FeO_x_ electrodes the Nyquist plot is a Warburg line with a slope close to 45° (slope = 55.6°), that is representative of diffusion-limited processes, for FeO_x_-CB electrode the Nyquist plot with a low frequency tale almost parallel to the imaginary axis (slope = 81.1°) describes a capacitive element. This enhancement confirms a remarkable improvement in charge (ions and electrons) diffusion for the nanocomposite. The uncompensated resistance that is evaluated at the highest frequency, R_u_, of FeO_x_-CB has not changed compared with that of FeO_x_ (R_u_ ~2.5 Ω) because this value is strictly controlled by the solution resistance and cell geometry which were the same for all the experiments.

**Figure 5:**
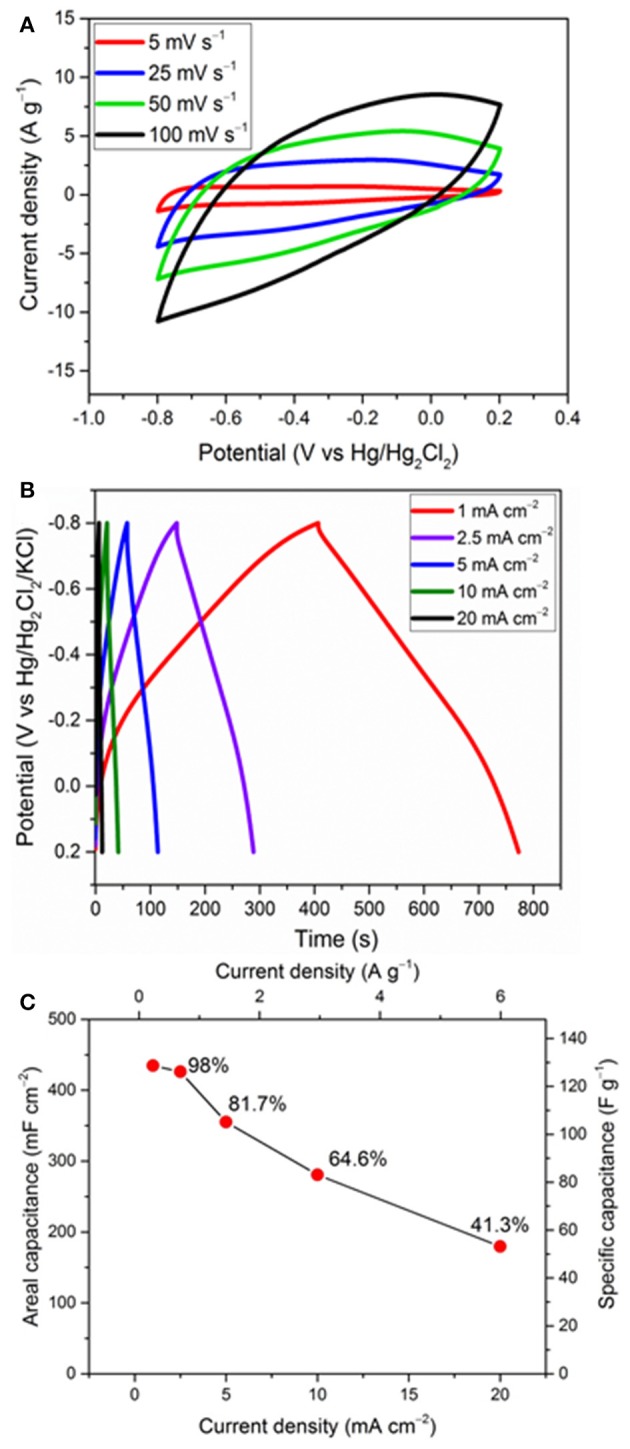
Rate performance: effect of different scan rates **(A)** and current densities **(B)** on the FeO_x_-CB obtained at 300°C with 18 wt.% carbon black, and the corresponding capacitance retention **(C)**.

Figure 5 exhibits the rate performance of the FeO_x_-CB, which is a crucial parameter in the evaluation of the supercapacitor electrodes. Figure 5A shows the effect of potential scan rate on the CVs of the FeO_x_-CB synthesized with 10 mg of CB (18 wt.% CB) at 300°C. The redox peaks are no longer seen at relatively high scan rates. However, it is reasonable to assume that the charge is stored dominantly through one or two electron transfer reactions between Fe^II^ and Fe^III^ (Xie et al., 2016). The CVs show a fair symmetry vs. potential axis at scan rates below 50 mV s^−1^, but they lose the symmetry at higher scan rates, which is due to the increasing limitation on ion diffusion in the electrode pores and the electron transfer within the electrode.

Figure 5B shows the GCD cycles of an FeO_x_-CB electrode with mass loading of 3.4 g cm^−2^ from 0.2 to −0.8 V at different current densities. The charge and discharge curves are symmetric with a coulombic efficiency of 91%, and the pseudocapacitive response holds in the same potential region of 0 to −0.8 V as that in the CVs. At 1 mA cm^−2^ the GCD curves do not show a noticeable ohmic drop. At 2.5 mA cm^−2^ the ohmic drop is 42 mV (Figure S3) which is fairly small and can be attributed to the facile electron migration and ion diffusion in the nanocomposite. The areal and specific capacitance and the corresponding capacitance retention at different current densities are shown in Figure 5C. The areal capacitance for FeO_x_-CB is 435 and 180 mF cm^−2^ at the current density of 1 and 20 mA cm^−2^, respectively, showing a capacitance retention of 41.3%. It has to be noted that at the highest current, which corresponds to 6 A g^−1^, the specific capacitance is 54 F g^−1^, a still high value which demonstrates the good performance of the proposed material.

Table 1 compares the specific capacitance of the FeO_x_-CB with some of the best iron oxide-based electrodes prepared by various methods. A specific capacitance of 128 F g^−1^ at a current density of 0.3 A g^−1^ with a high loading mass of 3.4 mg cm^−2^ make the FeO_x_-CB a competitive material that has the advantage of being produced by a low-cost, and sustainable method.

**Table 1:** Comparison of the FeO_x_-CB electrode features with iron oxide-based materials reported in the literature.

	**Synthesis method**	**Areal capacitance [mF cm ^**−2**^]**	**Specific capacitance [F g ^**−1**^]**	**Mass loading [mg cm^**−2**^]**	**Electrolyte**
Fe_3_O_4_ nanospheres (Aparna et al., 2018)	Solvothermal	–	101@2mV s^−1^	0.3	3 M KOH
Fe_3_O_4_/RGO (Yan et al., 2015)	Electrostatic	–	193@0.3 A g^−1^	–	6 M KOH
FeO_x_/RGO (Gao et al., 2014)	Benzyl alcohol	–	126@5mV s^−1^	–	1 M Na_2_SO_4_
Fe_2_O_3_ nanorods (Lu et al., 2014)	Hydrothermal	277.3@10 mV s^−1^	64.5@10mV s^−1^	4.3	1 M LiCl
Fe_3_O_4_/n-doped graphene (Liu et al., 2016)	Solvothermal	–	274@1 A g^−1^	2	2 KOH
Fe_x_O_y_-f-RGO (Rebuttini et al., 2015)	Solvothermal	–	79.7@20 mV s^−1^	**–**	1 M Na_2_SO_4_
Fe_2_O_3_ nanotubes (Yang et al., 2014)	Hydrothermal	180.4@1 mA cm^−2^	257.8@1.4 A g^−1^	0.7	5 M LiCl
Fe_3_O_4_/RGO (Lalwani et al., 2017)	Hydrothermal	–	63.5 @1 A g^−1^	0.3–0.5	H_2_SO_4_
Iron oxide/RGO (Wang et al., 2016)	Electrodeposition	406.5@10 mV s^−1^	–	0.15	5 M LiCl
FeO_x_-CB	FTA	408@0.3 A g^−1^	128@0.3 A g^−1^	3.4	1 M Na_2_SO_4_

**Figure 6:**
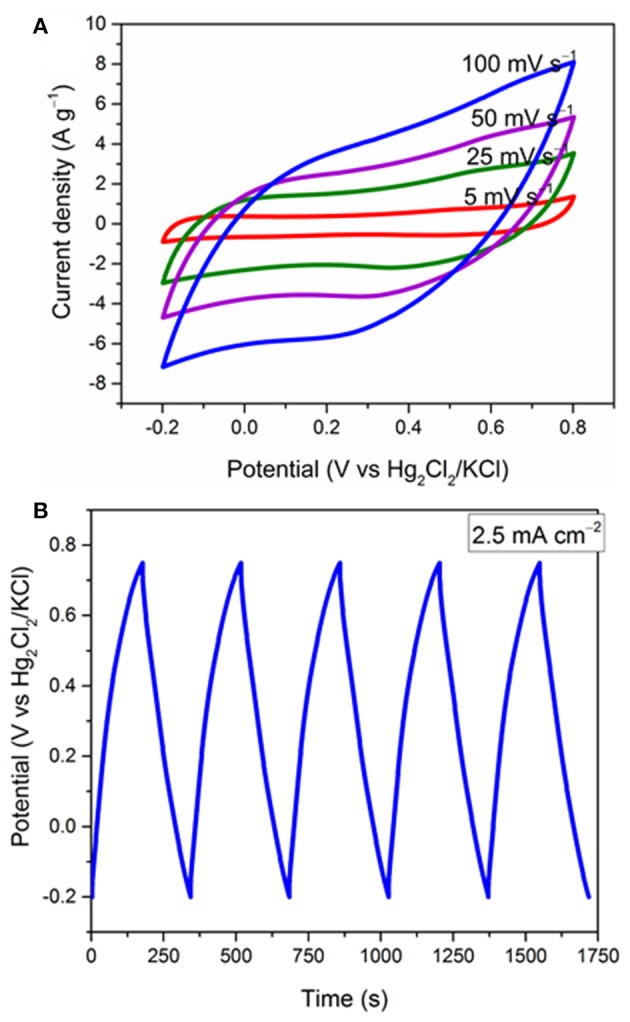
Electrochemical study of the a-Mn_3_O_4_/Ni foam (mass loading 2.5 mg cm^−2^). **(A)** effect of different scan rates on the CVs and **(B)** the GCD cycles at 2.5 mA cm^−2^.

Hausmannite (Mn_3_O_4_) was also coated *in situ* on Ni foam using a similar FTA method and investigated as the positive electrode by the CV and GCD techniques. For the Mn_3_O_4_/Ni foam, interestingly, the current density increases during the first 200 CV cycles (Figure S4). This effect has been also observed by Lokhande et al. and other groups for voltammetric cycling of Mn_3_O_4_ in alkali sulfate solutions, and it was attributed to the phase transformation of hausmanite (Mn_3_O_4_) to birnessite (MnO_2_) (Dubal et al., 2010a,b; Komaba et al., 2012). Therefore, an XRD of the Mn_3_O_4_/Ni foam after 200 cycles was recorded (Figure S5). The XRD pattern does not show any peak for MnO_2_; however, the Mn_3_O_4_ peaks are significantly less intense; therefore, the activation processes can be attributed to amorphisation during bulk sodium intercalation/deintercalation. This process is assisted by the intrinsic hydrophilicity of Mn_3_O_4_ (contact angle of 2–8°; Kulkarni et al., 2017) and the presence of carbon-oxygen groups. Figure 6A shows the effect of different scan rates on the electrochemical performance of a-Mn_3_O_4_ (activated by 200 cycles) from −0.2 to 0.8 V. It shows a semi-rectangular shape with a little distortion at high scan rates. Figure 6B shows the GCD cycles of the same electrode in the same potential window at 2.5 mA cm^−2^. The charge and discharge curves show a good symmetry with a coulombic efficiency of 98.4% and an ohmic drop of 82 mV measured from the discharge curves (Figure S6). The a-Mn_3_O_4_/Ni foam electrode exhibites a high areal capacitance of 480 mF cm^−2^. It is worth noting that the CVs and galvanostatic plots are representative of faradaic reactions involving (multi) electron transfer between Mn^4+^ and Mn^3+^ coupled with the deintercalation/intercalation of Na^+^ ions that is dominantly responsible for the charge storage of the material (Guillemet et al., 2012; Brousse et al., 2015; Kong et al., 2016; Costentin et al., 2017). Such good electrochemical performance of the a-Mn_3_O_4_ electrode was achieved without the need for adding CB in the composite. Therefore, in order to reduce the impact of electrochemically-inactive components on the total mass of supercapacitor, a-Mn_3_O_4_ electrodes were used without CB.

### Two-Electrode Studies

**Figure 7:**
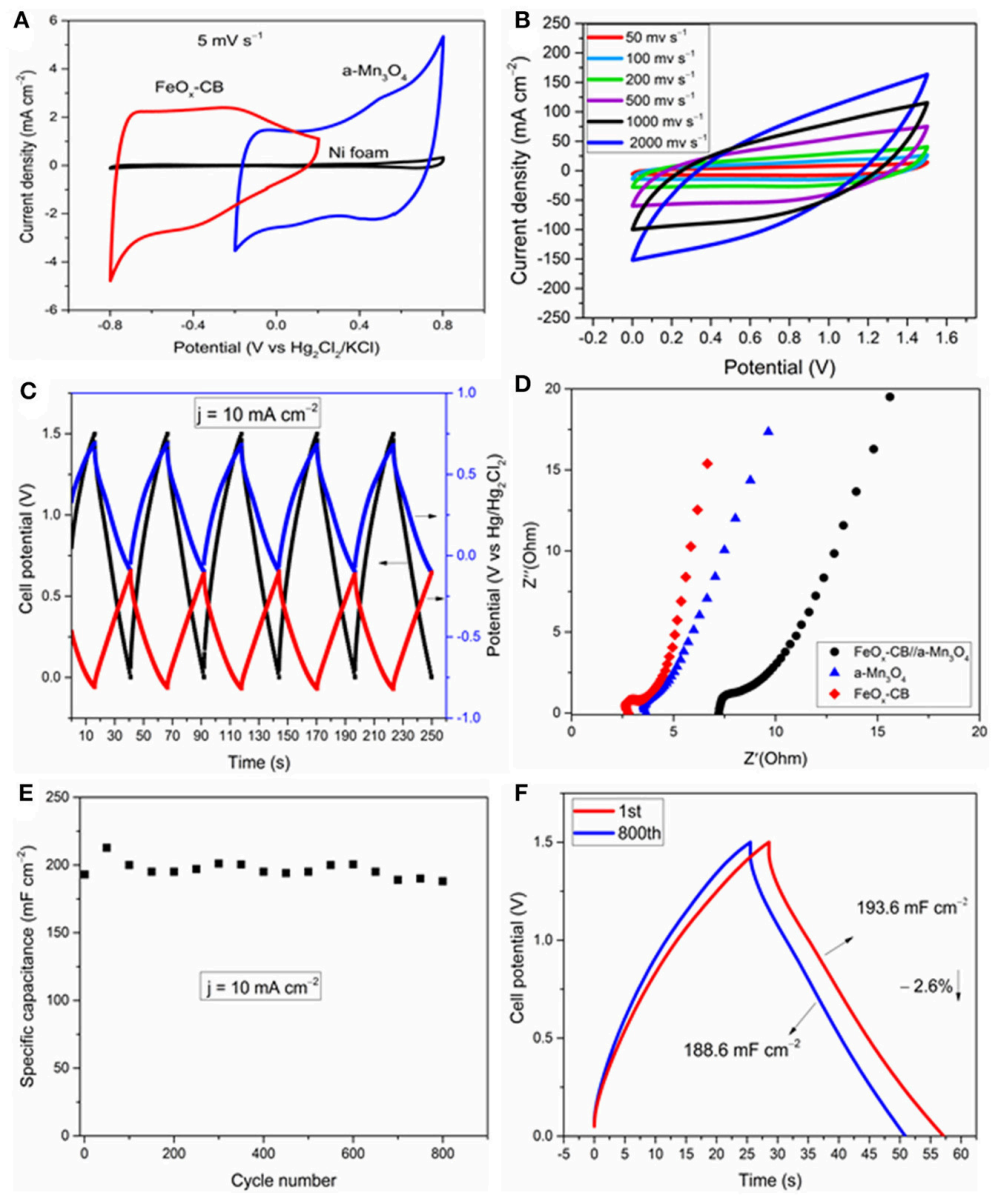
Electrochemical study of the FeO_x_-CB//a-Mn_3_O_4_ and the electrodes in the same cell. **(A)** CV cycles of the supercapacitor electrodes at 5 mV s^−1^, effect of scan rate **(B)**, GCD cycles at 10 mA cm^−2^: the black curve represents the cell voltage (left axis), the blue and red curves are the postive and negative electrode potentials (right axis) **(C)**, Nyquist plots **(D)**, areal capacitance vs. cycle number **(E)**, and comparison of the 1st and 800th cycle **(F)** at 10 mA cm^−2^.

As indicated above, both EDLC and pseudocapacitve processes operate in these electrodes. A definitive, yet simple, diagnostic test to distinguish both charging processes in these electrodes is to examine the shapes of the low-scan rate CVs (Costentin et al., 2017). Figure 7A shows the CVs of the two electrodes and the pristine Ni foam at 5 mV s^−1^ in a typical electrochemical glass cell. The improvements observed in the CV current densities of the electrodes compared to the Ni foam are mainly due to pseudocapacitive processes as revealed by their mirror-like broad redox peaks vs. potential axis. Similar redox peaks for carbon-Fe_3_O_4_ composites in the potential range of 0 to −0.5 V vs. Hg/Hg_2_Cl_2_ in sodium sulfate solution have been also reported by other groups. The redox peaks are attributed to Fe^II^/Fe^III^ electron transfer reaction, and as observed here, they are significantly promoted by the carbon supports such as reduced graphene oxide (Rebuttini et al., 2015; Naderi et al., 2016; Li et al., 2018) and carbon black (Sayahi et al., 2014). For Mn_3_O_4_, the redox peaks between 0.4 and 0.6 V are attributed to the reversible redox reaction between tetrahedral [Mn^II^O_4_] and octahedral [Mn^III^O_6_] (Yeager et al., 2013), in contrast to MnO_2_ which does not show any obvious redox peaks (Wang et al., 2017). Therefore, it can be concluded that the redox reactions in Fe_3_O_4_ and Mn_3_O_4_ are facilitated by simultaneous presence of two oxidation states of M^2+^ and M^3+^. The potential for the FeO_x_-CB and a-Mn_3_O_4_ pseudocapacitive processes span in the ranges of 0/−0, 8 V and −0.2/0, 8 V vs. Hg/Hg_2_Cl_2_, respectively, that allows charging the supercapacitor at a voltage of 1.6 V at maximum.

The FeO_x_-CB and a-Mn_3_O_4_ were used as the negative and positive electrodes, respectively, to assemble an asymmetric supercapacitor (FeO_x_-CB//a-Mn_3_O_4_). Figure 7B shows the voltammetric response of the supercapacitor at different scan rates over 1.5 V. Remarkably, the supercapacitor does not show a significant deviation in its mirror-like CV up to a high scan rate of 1,000 mV s^−1^, suggesting a small equivalent series resistance of the supercapacitor. However, at a still higher scan rate of 2,000 mV s^−1^ the CVs show a severe distortion, mainly due to ohmic contributions of the electrodes. A comparison of the CVs of the supercapacitor with that of the electrodes presented in Figures 5A, 6A reveals that the charge-discharge rate performance has improved for the Swagelok supercapacitor cell. This can be explained considering that cell geometry is optimized. In the supercapacitor cell, electrodes are contacted by their rear side while for 3-electrode test they were connected by co-axially climping the Nickel foam. Also, in the supercapacitor, the two electrodes are stacked and their distance is very small, corresponding to the separator thickness (ca. 100 μm), which in turn makes any contribution from electrolyte to the impedance of the system negligible.

Figure 7C shows the GCD cycles of the supercapacitor at a high current density of 10 mA cm^−2^ with simultaneous monitoring of the electrode potentials. Both electrodes actively participate in the charge/discharge in their respective potential windows, and the charge-discharge cycles for the supercapacitor are fairly symmetric vs. the potential axis. A negligible ohmic drop, resulting from a small ESR, is detectable at the start of the discharge. The supercapacitor shows a remarkable areal capacitance of 196 mF cm^−2^ at the current density of 10 mA cm^−2^. The energy density and power density calculated at 10 mA cm^−2^ are 0.06 mW h cm^−2^ (0.01 Wh/kg) and 8.3 mW cm^−2^ (1.4 W/kg), respectively. The coulombic efficiency is 99.1%.

**Scheme 2:**
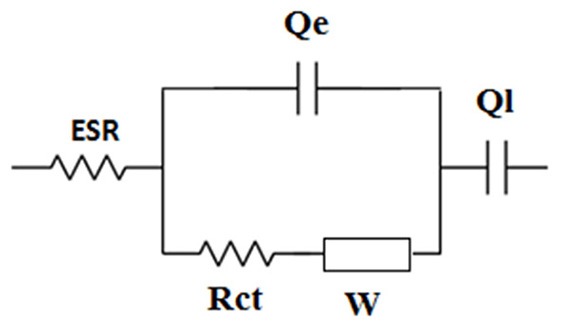
Equivalent circuit model used to fit the experimental Nyquist plots. ESR includes the electrolyte ionic resistance and electrode and current collector electronic resistances. Rct and Qe are the charge transfer resistance and the double layer capacitance. W is the Warburg element that describes diffusion/limited processes and Ql is the limit capacitance. Constant phase elecments Q are used to take into account the deviation by ideal capacitive behavior. Q = Y_0_(j ω)^−n^, where Y_0_ is the admittance and Q is a capacitance for *n* = 1.

EIS spectroscopy was used to disentangle the different underlying electrode processes and their effect on the performance of the supercapacitor. Figure 7D shows the Nyquist plots of the supercapacitor and the electrodes. The plots display three features: (i) small semicircles at the highest frequencies that are representative of the charge-transfer process, (ii) a middle frequency line with 45° slope, attributed to diffusion limited processes, and (iii) al low frequency line almost parallel to the imaginary axis that is related to the capacitive response of the system. The circuit model shown in Scheme 2 was used to fit the experimental nyquist plots. The ESR measured from the high frequency intercept of the Nyquist plots gives the real equivalent series resistance coupled with the capacitor (Yu et al., 2013). The ESR corresponds to the sum of each half-cell high frequency resistance Rei (ionic resistance of the electrolyte and the electrode electronic resistance). The ESR of 7 Ω was obtained for the supercapacitor, that agrees with the 7.2 Ω measured from the Bode plot when the phase shift is close to zero (Figure S7). It also agrees well with the ESR calculated from the Rei values for the electrodes (Rei(a-Mn_3_O_4_), Rei(FeO_x_-CB), the electrodes are equally spaced from the reference, therefore each term also includes ionic contribution from the electrolyte):

$$\begin{array}{l} \text{ESR}={\text{R}}_{\text{ei},\left(\text{MnOx}\right)}+{\text{R}}_{\text{ei},\left(\text{FeOx}-\text{CB}\right)}=3.7\Omega +2.5\Omega =6.2\Omega \end{array}$$

The ESR measured here for the supercapacitor reflects on first the solution resistance of the 1 M Na_2_SO_4_, and second the electrode resistance including the charge transfer resistance, Rct, which in turn depends on the interparticle electronic resistance and film-current collector contact resistance (Fic et al., 2012; Barsoukov and Macdonald, 2018). The Rct values measured for the FeO_x_-CB, a-Mn_3_O_4_, and the supercapacitor from the high frequency semicircle diameter of the Nyquist plots are 1.25, 0.15, and 1.7 Ω, respectively, indicating a more sluggish electron-transfer kinetics of the FeO_x_-CB compared with that of a-Mn_3_O_4_. However, comparison of the slopes of the lines in the low-frequency region reveals that the diffusional resistance of the supercapacitor is due more to the a-Mn_3_O_4_ electrode, that, therefore drives the overall response of the cell at the low frequency region. Nonetheless, a phase angle of −70° at 100 mHz for the supercapacitor (Figure S7) indicates that, though affected by diffusion, the process acting on the supercapacitor at low-frequency region approaches the limit capacitance. The areal capacitance of the supercapacitor was calculated to be 260 mF cm^−2^ at 100 mHz. In addition, the Bode plot of the supercapacitor in Figure S7 shows that at frequencies higher than 10 Hz the phaseshift angle tends to zero (i.e., between 0 and 10°); this indicates that for charge/discharge rates with a timescale lower than 0.1 s (i.e., logf = 1) the supercapacitor approaches the behavior of a resistor. This pattern agrees with that of the CVs in Figure 7B in which for a timescale lower than 0.8 s (i.e., 2,000 mV s^−1^) the supercapacitor approaches a resistive behavior.

Figures 7E,F show the cycling stability of the FeO_x_-CB//a-Mn_3_O_4_ supercapacitor over 800 GCD cycles. The specific capacitance of the supercapacitor after 800 cycles at 10 mA cm^−2^ reduced only by 2.6% from an initial value of 194 to 189 mF cm^−2^, showing a high cycling stability for the supercapacitor.

## Conclusion

If supercapacitors were to speak to the current demands in the electronics market, cost-effective and high-performance pseudocapacitor electrodes are indispensible. The use of benign materials and electrode processings that consume minimum amount of energy and materials are mandatory to meet the increasing market demand for supercapacitors and address the mounting environmental concerns. To this end, a fast annealing treatment of the Ni foam in a solution of the ethylene glycol was used to prepare high-performance pseudocapacitors without the need of any binder and energy, time, and cost demanding electrode lamination processes. Furthermore, it was shown that the *in-situ* added carbon black acts as a scaffold to disperse amorphous iron oxide particles; this resulted in the remarkable improvement of the electrochemical performance of FeO_x_-CB due to the promoted ion and electron transport. A manganese oxide electrode was also fabricated by a similar method and used as the positive electrode. Finally, the developed psuedocapacitor electrodes were used to assemble an asymmetric supercapacitor, and their true performance were proved promising. FTA can also be used to develop other metal-oxide electrodes for energetics and paves the way for a sustainable production of materials for energy storage.

## Author Contributions

Our submission is approved by all the authors and responsible authorities. FS and KM equally contributed to the research and result analysis and discussion. MG assisted in the synthesis and analysis of the materials.

### Conflict of Interest Statement

The authors declare that the research was conducted in the absence of any commercial or financial relationships that could be construed as a potential conflict of interest.
