# Supplementary material for: Toward Low-Cost and Sustainable Supercapacitor Electrode Processing: Simultaneous Carbon Grafting and Coating of Mixed-Valence Metal Oxides by Fast Annealing
Source: Front Chem. 2019 Feb 6;7:25. doi: 10.3389/fchem.2019.00025 (PMC6373437; doi:10.3389/fchem.2019.00025)
Supplement: Supplementary file 1 [file Data_Sheet_1.pdf]

Towards low-cost and sustainable supercapacitor electrode processing: simultaneous carbon grafting and coating of mixed-valence metal oxides by fast annealing

Keyvan Malaie,[a*] Francesca Soavi,[b*] and Mohammad Reza Ganjali[a]

[a] Center of Excellence in Electrochemistry, School of Chemistry, College of Science, University of Tehran, Enghelab St., 1417614411, Tehran, Tel: (0098) 9146352737

[b] Department of Chemistry "Giacomo Ciamician", Alma Mater Studiorum- Università di Bologna, Via Selmi 2, 40126, Bologna, Italy

1. Materials characterization

Table S1 EDS data for amorphous iron oxide-carbon black (FeOx-CB)

Elt	Line	Int	Error	K	Kr	W%	A%
**C**	Ka	140.4	6.7301	0.1825	0.1020	26.51	42.57
**O**	Ka	428.4	6.7301	0.2773	0.1550	37.26	44.92
**Fe**	Ka	480.0	0.8983	0.5401	0.3020	36.23	12.51
**Total**				1.0000	0.5590	100.00	100.00

W: Weight, A: Atomic, Int: Intensity

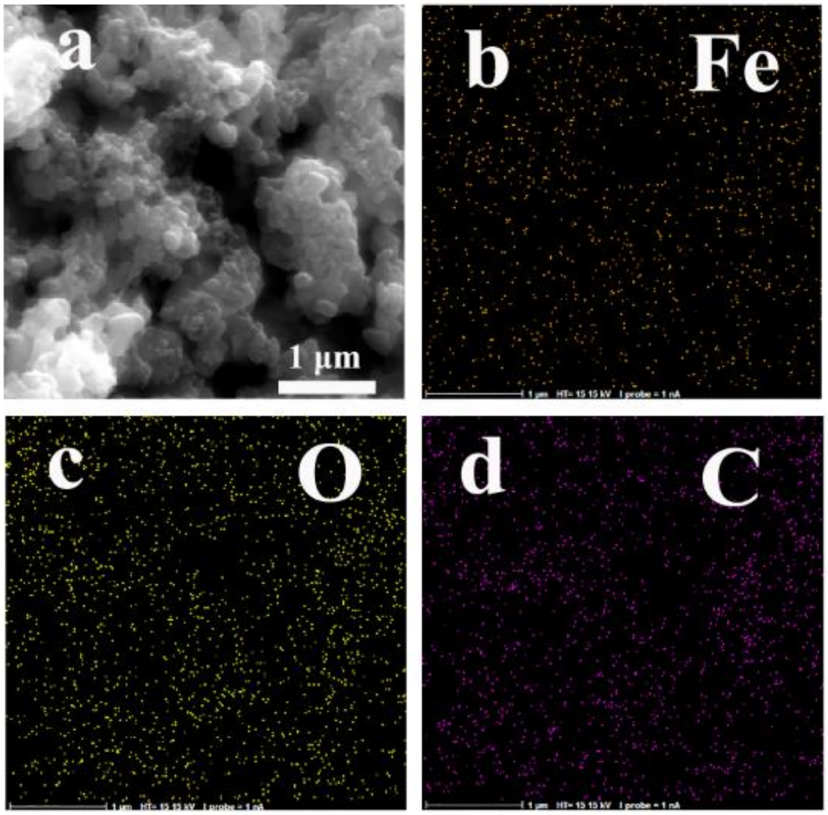

Fig. S1 FESEM and elemental mapping images of FeOx-CB

2. Electrochemical studies

2.1. Calculation of the oxidation state of iron oxide and its stochiometry

The oxidation state of iron and the stochiometry of iron oxide in the FeOx-CB sample were estimated according to the following procedure: First, 12 mg FeOx-CB powder was mixed in 1 mL of HCL (37%) by stirring for 5 minutes, and then, diluted by distilled water to a volume of 25 mL. This treatment leads to the dissolvation of the iron oxide according to the following equations:

$$Fe_3O_4 + 8HCL \longrightarrow FeCl_2 + 2FeCl_3 + 4H_2O \qquad \textbf{(1)}$$

The obtained dark-green suspention was sonicated for 5 minutes, and the carbon particles were decanted away. Finally, 5 ml of the solution was titrated potentiometrically by 0.025 M permanganate according to the following equation:

$$5Fe^{2+} + MnO^{4-} + 8H^{+} \longrightarrow Mn^{2+} + 5Fe^{3+} + 4H_2O \qquad \textbf{(2)}$$

For the titration experiment Ag/AgCl(KCl, 3M) and graphite rode were used as reference and indicator electrodes, respectively. Fig. S2 shows the titration curve and its differentiation curve.

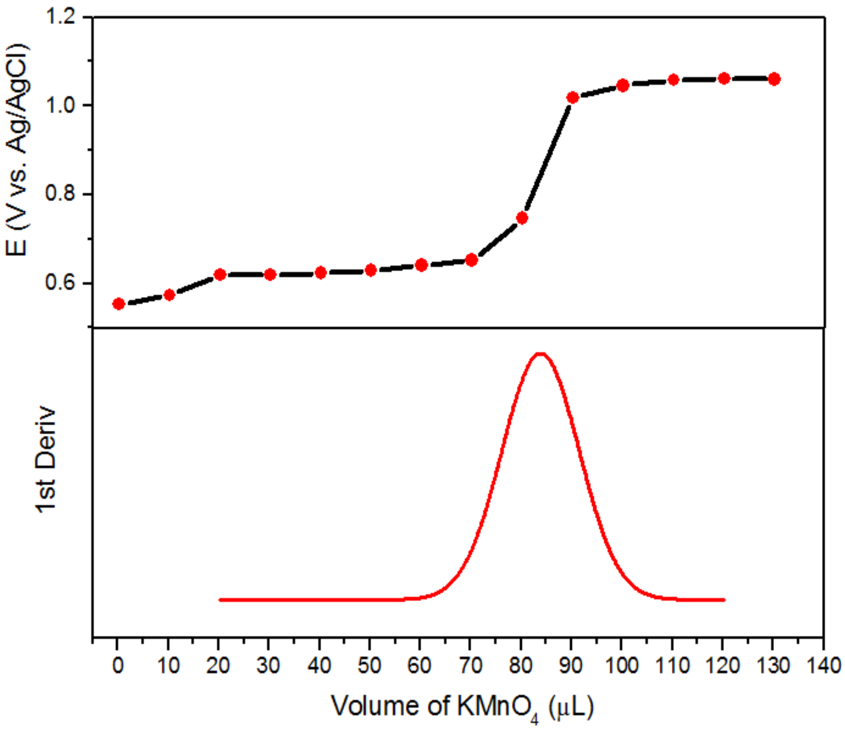

Fig. S2. Potentiometric redox titration of Fe(II) in the amorphous iron oxide-carbon black sample dissolved in HCL by 0.025 M

This titration showed that Fe(II) comprise 24.3 wt.% of the FeOx-CB sample (or 34.8 wt.% of the sample excluding total carbon). The amount of Fe(III) and O^{2-} ions in the compound $Fe^{II}_{34.8}Fe^{III}_{x}O_{y}$ can therefore be determined by solving two charge and mass balance equations:

Charge balance equation: $$\frac{34.8}{55.9}\times(+2)+\frac{x}{55.9}\times(+3)+\frac{y}{16}\times(-2)=0$$

Mass balance equation: $$34.8+x+y=100$$

x= 38.4% , y=26.8% ⟶ $Fe^{II}_{1} Fe^{III}_{1.1} O_{2.7}$ ⟶ $Fe^{II}_{2} Fe^{III}_{2} O_{5}$

2.2. Calculation of the performance parameters for the supercapacitor

The areal capacitance, energy density, and power density of the supercapacitor (FeOx-CB//MnOx) were calculated from the galvanostatic discharge curves. For a perfectly-linear GCD curve (or a linearly-fitted curve), the discharge curve can be treated as a right triangle. The slope of its diameter and the area inside it are a measure of the areal capacitance (C) and energy density (E), respectively, according to the following formulas:

$$C = \frac{j}{slope} \quad \textbf{(3)}$$

$$E = \frac{1}{2} \times (base) \times (height) = \frac{1}{2}(j \times t) \times (V) = \frac{1}{2} C V^2 \quad \textbf{(4)}$$

in which j the is current density (A cm^{-2}) and V is the working potential window in Volts. Therefore, the capacitance has a unit of F cm^{-2}. The energy density has a unit of C V cm^{-2} which is not practical for reporting; however, it can be easily converted to mW h cm^{-2} by multiplication to a factor of (1000/3600)[1]

The power density is simply the energy density in C V cm^{-2} divided by the discharge time, t_d (s):

[1] This results from multiplication of power by the discharge time in hour unit: $E = p \times \frac{t_d}{3600} = \frac{E}{t_d} \times \frac{t_d}{3600}$

$$P = \frac{E}{t_d} \quad \textbf{(5)}$$

Which gives power density in W cm^{-2}.

2.3. Electrochemical performance of the electrodes

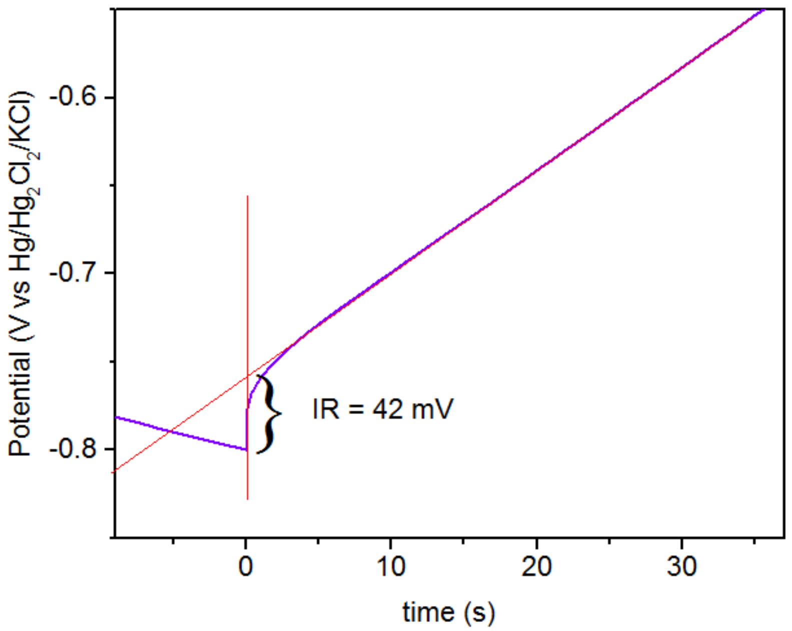

Fig. S3 IR drop measured from a discharge curve for FeOx-CB at the current density of 2.5 mA cm^{-2}

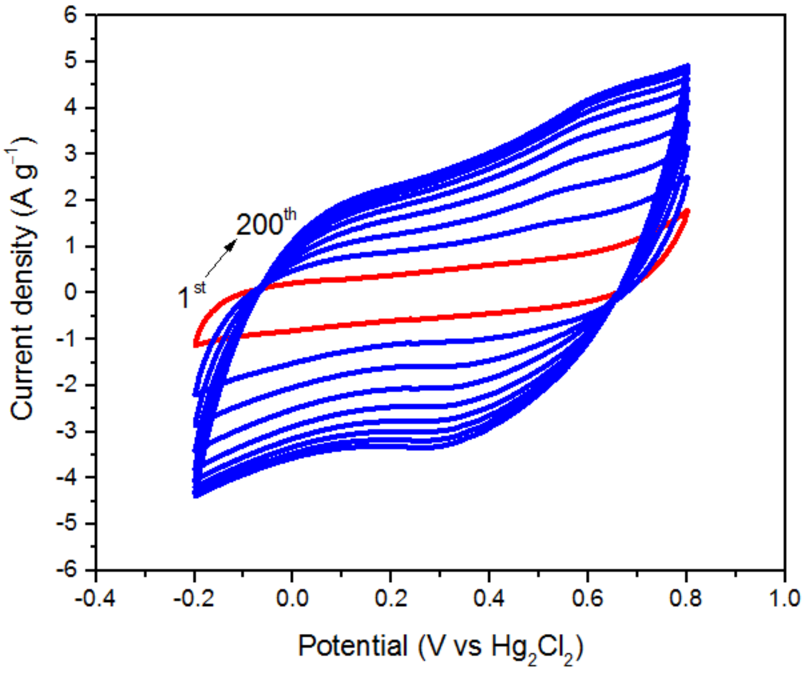

Fig. S4 Effect of voltammetric cycling on Mn_3O_4/Ni foam at 50 mV s^{-1} (mass loading 2.5 mg cm^{-2}).

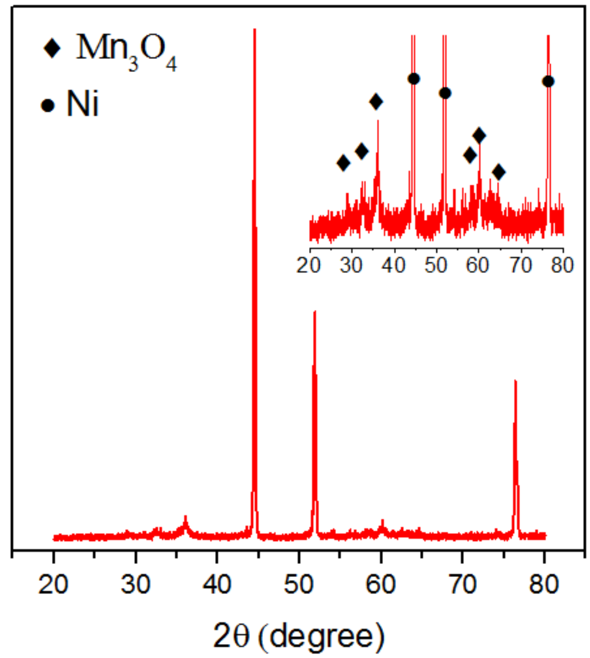

Fig. S5 XRD pattern of Mn_3O_4/Ni foam after running 200 cyclic voltammogram in 1 M Na_2SO_4. Inset: same XRD pattern magnified.

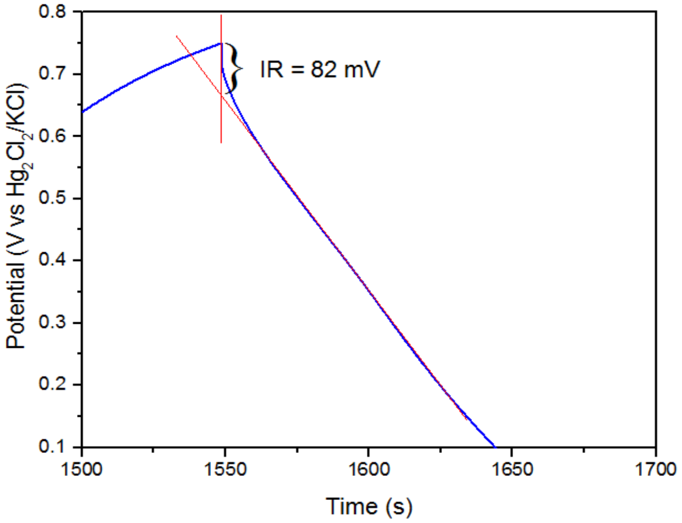

Fig. S6 The measurement of the IR drop for the Mn_3O_4/Ni foam electrode after 200 CV cycles at current density of 2.5 mA cm^{-2}

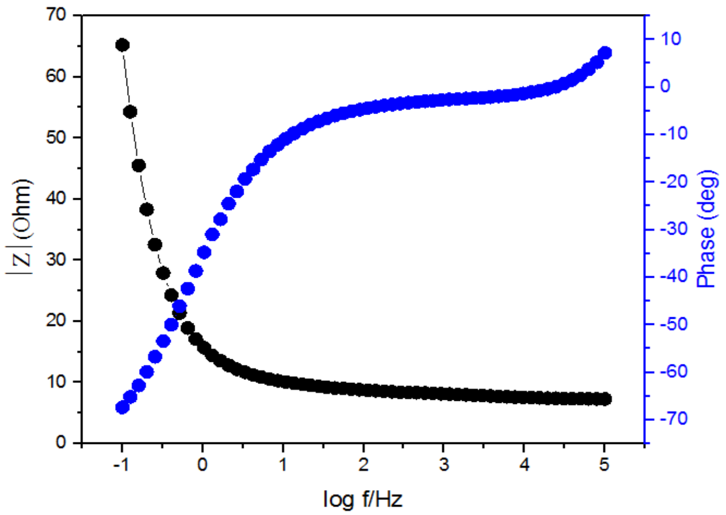

Fig. S7 Bode plot of the supercapacitor based on FeOx-CB//Mn_3O_4 in 1 M Na_2SO_4
